# Two new species of *Anacanthorus* (Monogenoidea, Dactylogyridae) parasitizing serrasalmid fish in Brazil

**DOI:** 10.1590/S1984-29612024007

**Published:** 2024-01-08

**Authors:** Augusto Leandro de Sousa Silva, Simone Chinicz Cohen, Michelle Daniele dos Santos-Clapp, Marilia Carvalho Brasil-Sato, Andréa Pereira da Costa, Marcia Cristina Nascimento Justo

**Affiliations:** 1 Programa de Pós-graduação em Ciência Animal, Laboratório de Multiusuários em Pesquisa da Pós-graduação – LAMP, Universidade Estadual do Maranhão – UEMA, São Luis, MA, Brasil; 2 Laboratório de Helmintos Parasitos de Peixes, Instituto Oswaldo Cruz, Fundação Oswaldo Cruz – Fiocruz, Rio de Janeiro, RJ, Brasil; 3 Laboratório de Biologia e Ecologia de Parasitos, Departamento de Biologia Animal, Instituto de Ciências Biológicas e da Saúde, Universidade Federal Rural do Rio de Janeiro – UFRRJ, Seropédica, RJ, Brasil; 4 Laboratório de Parasitologia e Doenças Parasitárias dos Animais – LPDPA, Universidade Estadual do Maranhão – UEMA, São Luís, MA, Brasil

**Keywords:** **:***Anacanthorus* spp., Dactylogyridae, Neotropical Region, Serrasalmidae, *Anacanthorus* spp., Dactylogyridae, Região Neotropical, Serrasalmidae

## Abstract

During studies on fish parasites, two new species of *Anacanthorus* were found parasitizing serrasalmid fishes, *Anacanthorus simpliciphallus* sp. n. from the hybrid *Piaractus mesopotamicus* x *Piaractus brachypomus* and *Anacanthorus brandtii* sp. n. from *Serrasalmus brandtii*. *Anacanthorus simpliciphallus* sp. n. resembles *Anacanthorus reginae* in the morphology of the male copulatory organ (MCO) and accessory piece but differs from *A. reginae* in terms of the smaller size of the accessory piece, which corresponds approximately half the size of the MCO and by the presence of a conspicuous metraterm, with a membranous terminal region. *Anacanthorus brandtii* sp. n. differs from *Anacanthorus scapanus* by the expansion of the accessory piece, from *Anacanthorus jegui* by the ratio MCO (male copulatory organ) /AP (accessory piece) and by the expansion of hook shank, from *Anacanthorus sciponophallus* and *A. reginae* by the ratio MCO/AP. *Anacanthorus brandtii* sp. n. can be distinguished from *A. reginae* and *A. simpliciphallus* sp.n. by the size of hooks which is similar in *A. reginae* and *A. simpliciphallus* sp. n. and dissimilar in *A. brandtii* sp. n. The two new species also differ from each other by the expansion of shank.

## Resumo

Durante estudos em parasitos de peixes, duas novas espécies de *Anacanthorus* foram encontradas parasitando peixes serrasalmideos, *Anacanthorus simpliciphallus* sp. n. do híbrido *Piaractus mesopotamicus* x *Piaractus brachypomus* e *Anacanthorus brandtii* sp. n. de *Serrasalmus brandtii. Anacanthorus simpliciphallus* sp. n. assemelha-se a *Anacanthorus reginae* na morfologia do órgão copulatório masculino (OCM) e peça acessória (PA), mas difere de *A. reginae* pelo tamanho menor da peça acessória, que corresponde a aproximadamente metade do tamanho do OCM e pela presença de um metraterma conspícuo, com uma região terminal membranosa. *Anacanthorus brandtii* sp. n. difere de *Anacanthorus scapanus* pela expansão da peça acessória, de *Anacanthorus jegui* pela proporção OCM / PA e pela expansão da haste do gancho, e de *Anacanthorus sciponophallus* e *A. reginae* pela proporção OCM / PA. *Anacanthorus brandtii* sp. n. pode ser distinguido de *A. reginae* e *A. simpliciphallus* sp. n. pelo tamanho dos ganchos que é similar em *A. reginae* e *A. simpliciphallus* sp. n. e dissimilar em *A. brandtii* sp. n. As duas espécies novas também diferem entre si pela expansão da haste do gancho.

## Introduction

Serrasalmidae, understood by fish known as piranhas and pacus, is a diverse family of freshwater fishes belonging to the order Characiformes, that is endemic throughout tropical and subtropical South America. *Piaractus mesopotamicus* (Holmberg, 1887) and *Piaractus brachypomus* (Cuvier, 1818), popularly known in Brazil as pacu and pirapitinga, respectively, are species characteristic of tropical waters and restricted to South America (Froese & Pauly, 2023). Crossing the female of *P. mesopotamicus* with the male of *P. brachypomus* results in the hybrid known as “patinga”, which has been gaining much ground in the Brazilian fish market (Ribeiro et al., 2016).

*Serrasalmus brandtii* Lutken, 1875 popularly known as white piranha and “pirambeba” is an endemic species of São Francisco River Basin (Britski et al., 1988; Jegú, 2003) and has a carnivorous feeding habit, being primarily piscivorous and secondarily insectivorous (opportunistic) (Pompeu & Godinho, 2003). The specimens generally inhabit lentic environments and are abundant in the Três Marias reservoir (Braga, 1975).

Dactylogyridae represents one of the most species-rich groups among helminths parasites of fishes (Boeger & Vianna, 2006; Cohen et al., 2013). Among all the genera of dactylogyrids, *Anacanthorus* Mizelle & Price, 1965 stand out as being highly diverse in species, distributed in a large number of host species among Neotropical freshwater fish (Cohen et al., 2013). Currently, this genus has 92 nominal species, among which 41 species (Table 1) parasitize Serrassalmidae fish, the most common host group for *Anacanthorus* spp*.* (Moreira et al., 2019). So far, species of *Piaractus* Eigenmann have been found to be parasitized by six species of Monogenoidea, among which *Anacanthorus* is the parasite genus most represented in this host genus: *Anacanthorus penilabiatus* Boeger, Husak & Martins, 1995; *Anacanthorus spathulatus* Kritsky, Thatcher & Kayton 1979; *Anacanthorus toledoensis* Leão, São Clemente & Cohen, 2015; *Mymarothecium ianwhittingtoni* Leão, São Clemente & Cohen, 2015; *Mymarothecium viatorum* Boeger, Piasecki & Sobecka, 2002; *Notozothecium janauachensis* Belmont-Jégu, Domingues, & Martins, 2004. These parasites have been recorded in Brazil and Peru (Kritsky et al., 1979; Boeger et al., 1995; Pamplona-Basilio et al., 2001; Martins et al., 2002; Cohen & Kohn, 2005, 2009; Lizama et al., 2007; Dinis-Vásquez et al., 2014; Leão et al., 2015, 2017; Oliveira & Tavares-Dias, 2016; Jerônimo et al., 2020; Moreira et al., 2019). During studies with fish parasites, a new species of *Anacanthorus* was found parasitizing the gills of a hybrid fish (*P. mesopotamicus x P. brachypomus*) that are commercialized in marketplace located in São Luís Island, Maranhão, Brazil, and another one in the endemic *S. brandtii* from São Francisco River and are described herein.

**Table 1:** *Anacanthorus* spp. parasites of Serrasalmidae from Neotropical Region. Scientific names of hosts are given according to accepted names in Froese & Pauly (2023).

MONOGENOIDEA	HOSTS	LOCALITIES	REFERENCES
*Anacanthorus amazonicus*Van Every & Kritsky, 1992	*Pristobrycon striolatus, Serrasalmus rhombeus, Serrasalmus altispinis, Serrasalmus* sp.	Bolívia, Brazil	Van Every & Kritsky (1992)
*Anacanthorus anacanthorus*Mizelle & Price, 1965	*Pygocentrus nattereri*	Brazil*	Mizelle & Price (1965)
*Anacanthorus beleophallus*Kritsky, Boeger & Van Every, 1992	*Pristobrycon eigenmanni*	Brazil	Kritsky et al. (1992)
*Anacanthorus brazilensis*Mizelle & Price, 1965	*P. nattereri*	Brazil^*^	Mizelle & Price (1965)
*Anacanthorus camposbacae*Morey, Aliano & Grandez, 2019	*Myloplus schomburgkii*	Peru	Morey et al. (2019)
*Anacanthorus carmenrosae*Morey, Aliano & Grandez, 2019	*M. schomburgkii*	Peru	Morey et al. (2019)
*Anacanthorus catoprioni*Kritsky, Boeger & Van Every, 1992	*Catoprion mento*	Brazil	Kritsky et al. (1992)
*Anacanthorus cinctus*Van Every & Kritsky, 1992	*P. striolatus, S. altispinis*	Brazil	Van Every & Kritsky (1992)
*Anacanthorus cladophallus*Van Every & Kritsky, 1992	*S. altispinis, S. spilopleura*	Brazil	Van Every & Kritsky (1992)
*Anacanthorus crytocaulus*Van Every & Kritsky, 1992	*S. altispinis, P. striolatus*	Brazil	Van Every & Kritsky (1992)
*Anacanthorus gravihamulatus*Van Every & Kritsky, 1992	*S. altispinis, S. rhombeus, P. eigenmanni, Serrasalmus* sp*.*	Bolívia, Brazil	Van Every & Kritsky (1992)
*Anacanthorus hoplophallus*Kritsky, Boeger & Van Every, 1992	*Myloplus rubripinnus*	Brazil	Kritsky et al. (1992)
*Anacanthorus jegui*Van Every & Kritsky, 1992	*Metynnis lippincottianus, P. eigenmanni, Pristobrycon* sp*., S. rhombeus, S. altispinis, Serrasalmus spilopleura, Serrasalmus* sp*.*	Bolívia, Brazil	Van Every & Kritsky (1992)
*Anacanthorus lasiophallus*Van Every & Kritsky, 1992	*P. striolatus*	Brazil	Van Every & Kritsky (1992)
*Anacanthorus lepyrophallus*Kritsky, Boeger & Van Every, 1992	*S. elongatus, S.altispinis, S. maculatus, S. marginatus, Serrasalmus* sp*.*	Brazil	Kritsky et al. (1992)
*Anacanthorus maltai*Boeger and Kritsky, 1988	*P. nattereri*	Brazil	Boeger & Kritsky (1988)
*Anacanthorus mastigophallus*Kritsky, Boeger & Van Every, 1992	*P. eigenmanni*	Brazil	Kritsky et al. (1992)
*Anacanthorus mesocondylus*Van Every & Kritsky, 1992	*S. spilopleura, Serrasalmus* sp*., P. eigenmanni, Pristobrycon* sp*.,*	Brazil	Van Every & Kritsky (1992)
*Anacanthorus myleusi*Moreira, Carneiro, Ruz & Luque, 2019	*M. schomburgkii*	Brazil	Moreira et al. (2019)
*Anacanthorus neotropicalis*Mizelle & Price, 1965	*P. nattereri*	Brazil*	Mizelle & Price (1965)
*Anacanthorus palamophallus*Kritsky, Boeger & Van Every, 1992	*P. eigenmanni*	Brazil	Kritsky et al. (1992)
*Anacanthorus paraspathulatus*Kritsky, Boeger & Van Every, 1992	*Mylossoma duriventris, M. aureum*	Brazil	Kritsky et al. (1992)
*Anacanthorus paraxaniophallus*Moreira, Carneiro, Ruz & Luque, 2019	*Serrasalmus maculatus, S. marginatus*	Brazil	Moreira et al. (2019)
*Anacanthorus pedanophallus*Kritsky, Boeger & Van Every, 1992	*M. rubripinnis*	Brazil	Kritsky et al. (1992)
*Anacanthorus penilabiatus*Boeger, Husak & Martins, 1995	*Colossoma macropomum, C. macropomum x Piaractus mesopotamicus, Piaractus brachypomus, P. mesopotamicus, P. brachypomus* x *P. mesopotamicus*	Brazil	Boeger et al. (1995), Pamplona-Basilio et al. (2001), Martins et al. (2002), Lizama et al. (2007), Cohen & Kohn (2009), Leão et al. (2017), Jerônimo et al. (2020)
*Anacanthorus periphallus*Kritsky, Boeger & Van Every, 1992	*S. altispinis, Serrasalmus* sp*.*	Brazil	Kritsky et al. (1992)
*Anacanthorus prodigiosus*Van Every &Kritsky, 1992	*S. elongatus, S. altispinis, S. rhombeus, Serrasalmus* sp*.*	Brazil	Van Every & Kritsky (1992)
*Anacanthorus ramosissimus*Van Every & Kritsky, 1992	*Serrasalmus elongatus*	Brazil	Van Every & Kritsky (1992)
*Anacanthorus reginae*Boeger & Kritsky, 1988	*P. nattereri*	Brazil, Peru	Boeger & Kritsky (1988), Iannacone & Luque (1993)
*Anacanthorus rondonensis*Boeger & Kritsky, 1988	*P. nattereri, S. rhombeus*	Brazil, Bolívia	Boeger & Kritsky (1988), Córdova & Pariselle (2007)
*Anacanthorus scapanus*Van Every & Kritsky, 1992	*S. spilopleura*	Brazil	Van Every & Kritsky (1992)
*Anacanthorus sciponophallus*Van Every & Kritsky, 1992	*S. altispinis, S. elongatus, S. maculatus, S.rhombeus, S. spilopleura, Serrasalmus* sp*.*	Bolívia, Brazil	Van Every & Kritsky (1992), Córdova & Pariselle (2007)
*Anacanthorus serrasalmi*Van Every & Kritsky, 1992	*S. altispinis, S. elongatus, S. rhombeus, Serrasalmus sp. Pristobrycon* sp*.,*	Brazil	Van Every & Kritsky (1992)
*Anacanthorus spathulatus*Kritsky, Thatcher & Kayton, 1979	*C. macropomum, C. macropomum x P. brachypomus, P. brachypomus*, *P. mesopotamicus*	Brazil, Peru, Venezuela	Kritsky et al. (1979), Aragot et al. (2002), Fischer et al. (2003) , Centeno et al. (2004), Lizama et al. (2007), Morais et al. (2009), Godoi et al. (2012), Santos et al. (2013), Soberon et al. (2014), Dias & Tavares-Dias (2015), Oliveira & Tavares-Dias (2016), Silva et al. (2022)
*Anacanthorus spinatus*Kritsky, Boeger & Van Every, 1992	*M. rubripinnus*	Brazil	Kritsky et al. (1992)
*Anacanthorus stachophallus*Kritsky, Boeger & Van Every, 1992	*P. nattereri*	Brazil, Peru	Kritsky et al. (1992), Iannacone & Luque, 1993
*Anacanthorus stagmophallus*Kritsky, Boeger & Van Every, 1992	*M.rubripinnis*	Brazil	Kritsky et al. (1992)
*Anacanthorus strongylophallus*Kritsky, Boeger & Van Every, 1992	*M. lippincottianus*	Brazil	Kritsky et al. (1992)
*Anacanthorus thatcheri*Boeger & Kritsky, 1988	*P. nattereri*	Brazil, Peru	Boeger & Kritsky (1988), Iannacone & Luque (1993)
*Anacanthorus toledeoensis*Leão, São Clemente & Cohen, 2015	*P. mesopotamicus*	Brazil	Leão et al. (2015)
*Anacanthorus xaniophallus*Kritsky, Boeger & Van Every, 1992	*P. eigenmanni, Pristobrycon* sp*.*	Brazil	Kritsky et al. (1992)

* Host obtained from Steinhart Aquarium, San Francisco, California

## Material and Methods

One hybrid specimen of *P. mesopotamicus x P. brachypomus* purchased from a fish market on São Luís Island, State of Maranhão, which had been brought to the market from a fish farm established in the municipality of Matinhas (3º05'13.5"S, 45º02'56"W) and 168 specimens of *S. brandtii* captured by local fishers in Três Marias Reservoir (18º12'59"S, 45º17'34"W), Upper São Francisco River, Minas Gerais State, Brazil and sent to the “Centro Integrado de Recursos Pesqueiros e Aquicultura (CIRPA)” of the “Companhia de Desenvolvimento dos Vales do São Francisco e Parnaíba (CODEVASF)” were examined for Monogenoidea. The gills were removed and placed in vials containing hot water (~65ºC) and were shaken. Absolute ethanol was added to reach a concentration of 70%. Monogenoids were picked from the sediment and from the gill arches with the aid of a stereoscopic microscope. Some specimens were mounted in Hoyer’s medium to study of the sclerotized parts and others were stained with Gomori’s trichrome and mounted in Canada balsam (Humason, 1979; Boeger & Vianna, 2006). The specimens were observed using an Olympus BX 41 microscope with phase contrast and Zeiss Axioskop 2 Plus microscope with differential interference contrast, both equipped with a camera lucida for drawings. All measurements are presented in micrometers, and the range is followed by the mean in parentheses and the number of specimens measured. Identification of the authors and nomenclatural acts for the taxon was in accordance with the guidelines provided in Article 50.1 and recommendation 50A of the International Code of Zoological Nomenclature (ICZN), which specifically pertains to authorship identity. The holotype and paratypes for each parasite species were deposited in the Helminthological Collection of the Oswaldo Cruz Institute (CHIOC), Rio de Janeiro, Brazil.

## Results

TAXONOMY

Class Monogenoidea Bychowsky, 1937

Subclass Polyonchoinea Bychowsky, 1937

Order Dactylogyridea Bychowsky, 1937

Family Dactylogyridae Bychowsky, 1933

Subfamília Anacanthorinae Mizelle & Price, 1965

*Anacanthorus* Mizelle & Price, 1965

**Figure 1:**
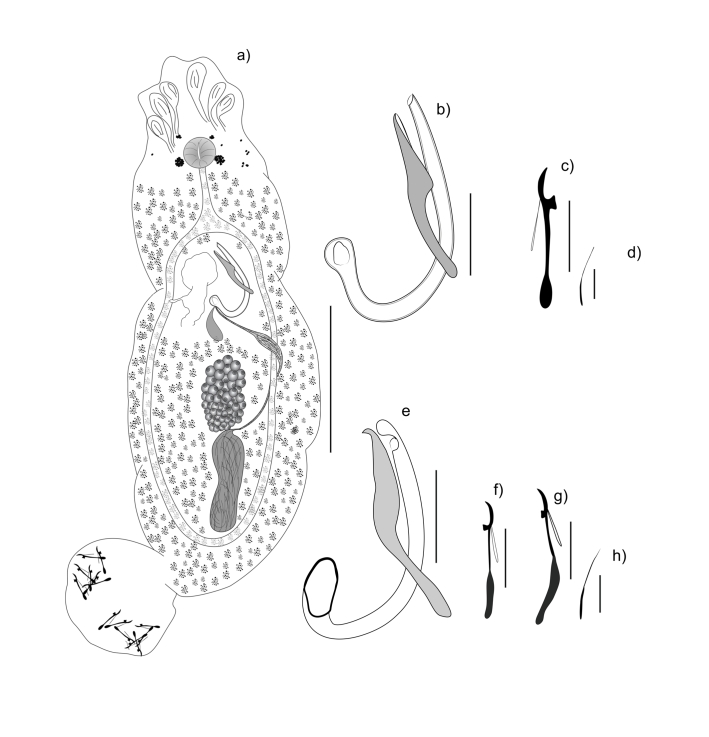
a-d: *Anacanthorus simpliciphallus* sp. n. parasite of hibrid *Piaractus mesopotamicus x Piaractus brachypomus.* (a): Total view, ventral (composite) (b): MCO (c) Hook (d) Hook 4A; e-h: *Anacanthorus brandtii* sp. n. parasite of *Serrasalmus brandtii*: (e): MCO (f) Hook pairs 1,5 (g) Hook pairs 2,4,6,7 (h) Hook 4A. Scale bars: (a) 100 µm (b, e) 20 µm (c, f, g) 10 µm (d, h) 5 µm.

**Figure 2:**
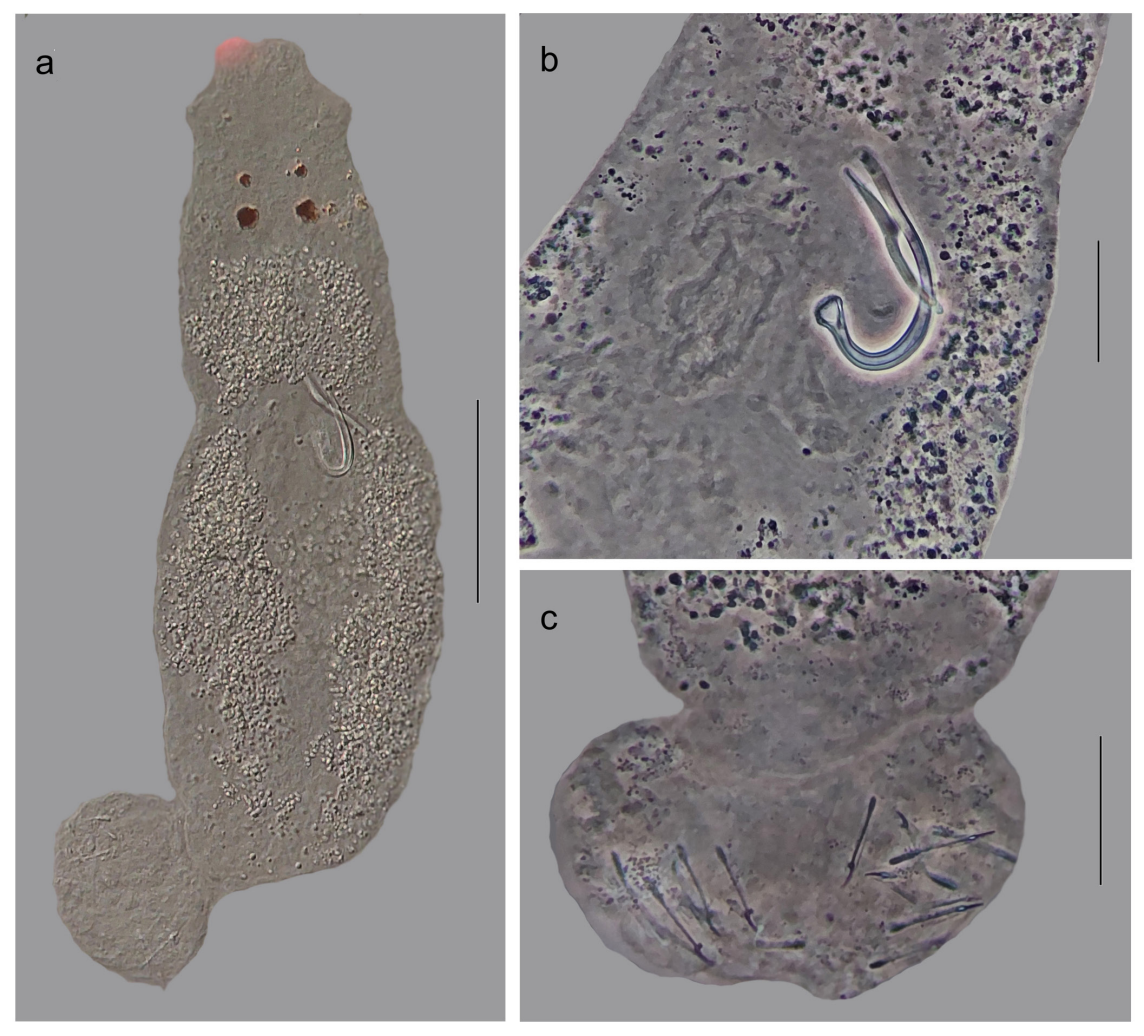
Light photomicrographs of *Anacanthorus simpliciphallus* sp. n. parasite of hybrid *Piaractus mesopotamicus* x *Piaractus brachypomus.* (a): Total view, ventral (b) Copulatory complex (c) Haptor. Scale bars. (a) 100 µm, (b) 30 µm; (c) 30 µm.

***Anacanthorus simpliciphallus***Silva, Cohen, Costa & Justo sp. n. (Figure 1a-d; Figure 2a-c).

Type host: Hybrid *Piaractus mesopotamicus x Piaractus brachypomus* (Characiformes, Serrasalmidae)

Site in host: Gill lamellae

Type-locality: Marketplace on São Luis Island, Maranhão state, host specimen obtained from a fish farm established in the municipality of Matinhas (3º05'13.5"S, 45º02'56" W).

Parasitological indexes: Total number of hosts: 1; total number of parasites: 7

Type-material: Holotype CHIOC 40268 a; Paratypes CHIOC 40268 b-g.

Etymology: The species name is from Latin (simplex=simples + phallus=penis) and refers to the morphology of the male copulatory organ.

DESCRIPTION: (Based on seven specimens: six mounted in Hoyer’s medium and one mounted in Gomori’s trichrome). Body elongated, fusiform, 262–550 (422, n= 5) long including the haptor, by 88–145 (123, n= 5) wide at the level of germarium. Two terminal, and two bilateral well developed cephalic lobes; three bilateral pairs of head organs. Two pairs of eyes equidistant, anterior pair smaller than posterior pair, slightly closer together than posterior pair; pairs slightly close to each other; accessory granules sparse in the cephalic region. Pharynx subspherical, 20 and 27 (n= 2) in diameter; long oesophagus. Two intestinal ceca confluent posterior to the gonads, lacking diverticula. Gonads overlapping; testis dorsal to germarium, 60–100 (86; n= 4) long, vas deferens looping intestinal caeca, single prostatic reservoir pyriform. Copulatory complex comprising male copulatory organ (MCO) and accessory piece (AP). MCO tubular, heavily sclerotized, J-shaped, with slightly sclerotized walls, base with smooth margin, 65–83 (73; n= 7) long. Accessory piece with a terminal flap, non articulated to MCO base, 37–45 (41; n= 7). Ratio MCO/AP 1:0.48-1:0.58 (1:0.55, n=7). Germarium 35 and 40 (n= 2) long by 40 and 45 (n= 2) wide. Metraterm conspicuous, with membranous terminal region. Genital pore and eggs not observed. Peduncle short. Haptor armed with 7 pairs of hooks (4 ventral, 3 dorsal), 2 pairs (1 dorsal, 1 ventral) of 4A's, 60–135 (93, n= 5) wide. Hooks similar in shape and size, each with truncate slightly depressed thumb, curved shaft, short point, shank proximal expansion 0.3 shank length, 20–24 (21; n= 20) long; filamentous hook (FH loop) delicate, extending as far as half of the shank. Similar 4A hooks, 9–12 (10; n= 10). Vitellaria dense, dispersed throughout the trunk, absent in the region of reproductive organs and copulatory complex.

**Remarks:***Anacanthorus simpliciphallus* sp. n. differs from all congeneric species mainly in terms of the morphology of the accessory piece. The new species resembles *Anacanthorus reginae* Boeger & Kritsky, 1988, in the morphology of the male copulatory organ (J-shaped) and in that the accessory piece is not articulated to the MCO base and has a terminal flap. Both species differs mainly with regard to the ratio between MCO and accessory piece [practically the same size (MCO 57–76; accessory piece 42–67) in *A. reginae x* accessory piece 50% the size (MCO 65–83; accessory piece 37–45) in *Anacanthorus simpliciphallus* sp. n. and with regard to the size of hooks (23–34 (28) in *A. reginae* and 20–24 (21) in the new species). Moreover, the new species can be differentiated from *A. reginae* in that it has a metraterm conspicuous, with a membranous terminal region.

**Figure 3:**
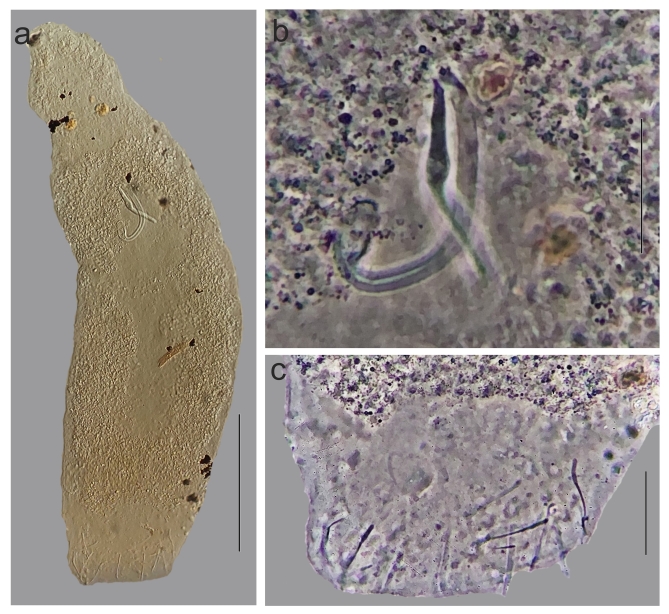
Light photomicrographs of *Anacanthorus brandtii* sp. n. parasite of *Serrasalmus brandtii.* (a): Total view, ventral (b) Copulatory complex (c) Haptor. Scale bars. (a) 100 µm, (b) 30 µm; (c) 20 µm.

***Anacanthorus brandtii***Santos-Clapp, Cohen, Justo & Brasil-Sato sp. n (Figure 1e-h; Figure 3a-c)**.**

Type host: *Serrasalmus brandtii* Lütken, 1875 (Characiformes, Serrasalmidae)

Site in host: Gill lamellae

Type-locality: Três Marias Reservoir (18º12'59" S, 45º17'34" W), Upper São Francisco River, Minas Gerais State.

Parasitological indexes: Total number of hosts: 145; total number of parasites: 142

Type-material: Holotype CHIOC 40263 a; Paratypes CHIOC 40263 b; 40264; 40265; 40266 a,b; 40267 a, b.

Etymology: The new species is named after the specific epithet of the host, *Serrasalmus brandtii*

DESCRIPTION: (Based on 30 specimens mounted in Hoyer’s medium). Body elongated, fusiform, 295–595 (433, n= 12) long including the haptor, by 100–165 (130, n= 12) wide at the level of germarium. Two terminal, and two bilateral cephalic lobes; three bilateral pairs of head organs. Two pairs of eyes, anterior pair smaller than posterior pair, slightly closer together than posterior pair; accessory granules distributed in the cephalic region. Pharynx subspherical, long oesophagus. Intestinal ceca lacking diverticula. Gonads overlapping; testis dorsal to germarium, 70–130 (84; n=6) long, vas deferens looping intestinal caeca, prostatic reservoir not observed. Copulatory complex comprising male copulatory organ (MCO) and accessory piece. MCO as a J-shaped tube, with slightly sclerotized walls, expanded base with smooth margin, 60–78 (70; n=13) long. Accessory piece with a midlength expansion extended to distal region, non articulated to MCO base, 38–45 (41; n= 13). Ratio MCO/AP 1:0.58–1:0,63 (1:0.6, n=13) Germarium, metraterm, genital pore and eggs not observed. Peduncle inconspicuous. Haptor armed with 7 pairs (4 ventral, 3 dorsal) of hooks, 2 pairs (1 dorsal, 1 ventral) of 4A's, 68–120 (94, n=12) wide. Hooks similar in shape, each with truncate slightly depressed thumb, curved shaft, short point, pairs 1,5, 20–24 (22, n=13) long, proximal expansion 0.4 shank length, pairs 2–4,6,7, 28–32 (30, n=13) long, proximal expansion 0.6 shank length; Filamentous hook (FH loop) delicate, extending until up to half of the shank. Similar 4A hooks, 10–15 (12; n=6). Vitellaria dense, dispersed throughout the trunk, absent in the region of reproductive organs and copulatory complex.

Remarks: *Anacanthorus brandtii* sp. n. is closely related to species previously described from *Serrasalmus* spp. as *Anacanthorus scapanus*, *Anacanthorus jegui*, *Anacanthorus sciponophallus*, *A. reginae* and *A. simpliciphallus* sp. n. by the morphology of copulatory complex. The new species differs from *A. scapanus* by the expansion of the accessory piece (subterminal in *A. scapanus vs* midlength expansion in the *A. brandtii* sp. n.), from *A. jegui* by the ratio MCO/AP (MCO 48 and AP 39 in *A. jegui* and MCO 70 *vs* AP 41 in the new species) and by the expansion of hook shank (0.3 and 0.4 in *A. jegui vs* 0.4 and 0.6 in *A. brandtii* sp. n.). The new species can be differentiated from *A. sciponophallus* and *A. reginae* by the ratio MCO/AP (MCO 76–82 and AP 74–79 in *A. sciponophallus* from different hosts, MCO 67 and AP 59 in *A. reginae vs* MCO 70 and AP 41 in the new species). The new species can also be distinguished from *A. reginae* and *A. simpliciphallus* sp. n. by the size of hooks which is similar in *A. reginae* and *A. simpliciphallus* sp. n. *vs* dissimilar in *A. brandtii* sp. n. The two new species also differ by the expansion of shank (0.3 in *A. simpliciphallus* sp. n. *vs* 0.4 and 0.6 in *A. brandtii* sp. n.).

## Discussion

The new species are allocated in *Anacanthorus* because they possess a bilobed haptor with 7 pairs of hooks and 2 pairs of reduced hooks (4A’s), lacking anchors and bars, have tandem or slightly overlapping gonads, post-ovarian testis, modified (thickened or sclerotized) distal uterine wall or metraterm and vagina is absent (Kritsky et al., 1979, 1992).

*Anacanthorus* species are exclusively parasites of Neotropical characiforms, and so far, 19 species have been reported from Bryconidae, 8 from Erythrinidae, 21 from Triportheidae, and 44 from Serrasalmidae. Kritsky & Thatcher (1974) described *Anacanthorus colombianus* Kritsky & Thatcher, 1974 from *Salminus affins* Steindachner, 1880 and also reported its presence in *Oreochromis mossambicus* (Peters, 1852), a cichlid fish within the order Cichliformes. According to these authors, this latter occurrence was apparently accidental. Given the absence of further records in this order, it is considered that *Anacanthorus* spp. is specific to characiform fishes. Species of this genus have been found in five countries in the Neotropical Region (Brazil, Bolivia, Colombia, Peru, and Venezuela), and Brazil stands out as the country with the largest number of occurrences (156) (Boeger et al., 2023).

The sclerotized structures such as the copulatory complex and hooks of *Anacanthorus* species appear to have a high specificity in terms of morphology with regard to the host family level (Santos et al., 2019). *Anacanthorus* species that parasitize members of the family Serrasalmidae present the characteristic of a J-shaped MCO, an accessory piece that is not articulated to the MCO, hooks with truncated thumb and a shank with proximal dilation (Boeger & Kritsky, 1988; Kritsky et al., 1992; Van Every & Kritsky, 1992). The finding of two new species of *Anacanthorus* in serrasalmid hosts presenting morphological characteristics similar to those previously described on these hosts (Table 1) confirms that the lineages of the parasites from serrasalmid hosts shared those features.

## References

[B001] Aragot W, Morales G, León E, Pino L, Guillén A, Silva M (2002). Patologías asociadas a monogéneos branquiales en cachama bajo cultivo. Vet Trop.

[B002] Boeger WA, Cohen SC, Domingues MV, Justo MCN, Pariselle A (2023). Monogenoidea. Catálogo Taxonômico da Fauna do Brasil.

[B003] Boeger WA, Husak WS, Martins ML (1995). Neotropical Monogenoidea. 25. *Anacanthorus penilabiatus* n. sp. (Dactylogyridae, Anacanthorinae) from *Piaractus mesopotamicus* (Osteichthyes, Serrasalmidae), cultivated in the state of São Paulo Brazil. Mem Inst Oswaldo Cruz.

[B004] Boeger WA, Kritsky DC (1988). Neotropical Monogenea. 12. Dactylogyridae from *Serrasalmus nattereri* (Cypriniformes, Serrasalmidae) and aspects of their morphologic variation and distribution in the Brazilian Amazon. Proc Helminthol Soc Wash.

[B005] Boeger WA, Vianna RT, Thatcher VE (2006). Aquatic biodiversity in Latin America.

[B006] Braga RA (1975). Ecologia e etologia das piranhas do nordeste do Brasil (Pisces – Serrasalmus Lacépède, 1803)..

[B007] Britski HA, Sato Y, Rosa ABS (1988). Manual de identificação de peixes da região de Três Marias (com chaves de identificação para os peixes da Bacia do São Francisco).

[B008] Centeno L, Silva-Acuña A, Silva-Acuña R, Pérez JL (2004). Fauna ectoparasitaria asociada a *Colossoma macropomum* y al híbrido de *C. macropomum* x *Piaractus brachypomus*, cultivados en el Estado Delta Amacuro, Venezuela. Bioagro.

[B009] Cohen SC, Justo MCN, Kohn A (2013). South American Monogenoidea parasites of fishes, amphibians and reptiles..

[B010] Cohen SC, Kohn A (2005). A new species of *Mymarothecium* and new host and geographical records for *M. viatorum* (Monogenea: Dactylogyridae), parasites of freshwater fishes in Brazil. Folia Parasitol (Praha).

[B011] Cohen SC, Kohn A (2009). On Dactylogyridae (Monogenea) of four species of characid fishes from Brazil. Check List.

[B012] Córdova L, Pariselle A (2007). Monogenoidea en *Serrasalmus rhombeus* (Linnaeus 1766) de la Cuenca Amazónica Boliviana. Rev Peru Biol.

[B013] Dias MKR, Tavares-Dias M (2015). Seasonality affects the parasitism levels in two fish species in the eastern Amazon region. J Appl Ichthyology.

[B014] Dinis-Vásquez N, Soplín-Bosmediano M, Pizango-Paima E, Chu-Koo F, Verdi-Olivares L (2014). Índices parasitarios en larvas, post larvas y alevinos de *Piaractus brachypomus* “paco” en relación a los factores ambientales. Cienc Amaz.

[B015] Fischer C, Malta JCO, Varella AMB (2003). A fauna de parasitas do tambaqui, *Colossoma macropomum* (Cuvier, 1818) (Characiformes: Characidae) do médio rio Solimões, Estado do Amazonas (AM) e do baixo rio Amazonas, Estado do Pará (PA), e seu potencial como indicadores biológicos. Acta Amaz.

[B016] Froese R, Pauly D (2023). FishBase. Version (2/2023).

[B017] Godoi MMIM, Engracia V, Lizama MLAP, Takemoto RM (2012). Parasite-host relationship between the tambaqui (*Colossoma macropomum* Cuvier 1818) and ectoparasites, collected from fish farms in the City of Rolim de Moura, State of Rondônia, Western Amazon, Brazil. Acta Amaz.

[B018] Humason GL (1979). Animal tissue techniques..

[B019] Iannacone J, Luque JL (1993). New records on helminths parasitic on Peruvian Amazonian. Fishes (Osteichthyes). Rev Biol Trop.

[B020] Jegú M, Reis RE, Kullander SO, Ferraris CJ (2003). Check list of the freshwater fishes of South and Central America..

[B021] Jerônimo GT, Ventura AS, Pádua SB, Porto EL, Ferreira LC, Ishikawa MM (2020). Parasitological assessment in hybrids Serrasalmidae fish farmed in Brazil. Rev Bras Parasitol Vet.

[B022] Kritsky DC, Boeger WA, Van Every LR (1992). Neotropical Monogenoidea. 17. *Anacanthorus* Mizelle and Price, 1965 (Dactylogyridae, Anacanthorinae) from characoid fishes of the central Amazon. J Helminthol Soc Wash.

[B023] Kritsky DC, Thatcher VE, Kayton RJ (1979). Neotropical Monogenoidea. 2. The Anacanthorinae Price, 1967, with the proposal of four new species of *Anacanthorus* Mizelle & Price, 1965 from Amazonian fishes. Acta Amaz.

[B024] Kritsky DC, Thatcher VE (1974). Monogenetic trematodes (Monopisthocotylea: Dactylogyridae) from freshwater fishes of Colombia, South America. J Helminthol.

[B025] Leão MSL, Justo MCN, Bueno GW, Cohen SC, São Clemente SC (2017). Parasitism by Monogenoidea in *Piaractus mesopotamicus* (Characiformes, Characidae) cultivated in Paraná River (Brazil). Braz J Biol.

[B026] Leão MSL, São Clemente SC, Cohen SC (2015). *Anacanthorus toledoensis* n. sp. and *Mymarothecium ianwhittingtoni* n. sp. (Dactylogyridae: Monogenoidea) parasitizing Cage-Reared *Piaractus mesopotamicus* (Characiformes, Characidae) in the State of Paraná, Brazil. Comp Parasitol.

[B027] Lizama MAP, Takemoto RM, Ranzani-Paiva MJT, Ayrosa LMS, Pavanelli GC (2007). Relação parasito-hospedeiro em peixes de pisciculturas da região de Assis, estado de São Paulo, Brasil. 2. *Piaractus mesopotamicus* (Holmberg, 1887). Acta Sci Biol Sci.

[B028] Martins ML, Moraes FR, Miyazaki DMY, Brum CD, Onaka EM, Fenerick J (2002). Alternative treatment for *Anacanthorus penilabiatus* (Monogenea: Dactylogyridae) infection in cultivated pacu, *Piaractus mesopotamicus* (Osteichthyes: Characidae) in Brazil and its haematological effects. Parasite.

[B029] Mizelle JD, Price CE (1965). Studies on monogenetic trematodes. XXVIII. Gill parasites of the piranha with proposal of *Anacanthorus* gen. n. J Parasitol.

[B030] Morais AM, Varella AMB, Villacorta-Correa MA, Malta JCO (2009). A fauna de parasitos em juvenis de tambaqui *Colossoma macropomum* (Cuvier, 1818) (Characidae: Serrasalminae) criados em tanques-rede em Lago de várzea da Amazônia Central. Biol Geral Exper.

[B031] Moreira J, Carneiro JS, Ruz EJH, Luque JL (2019). New species and records of *Anacanthorus* (Monogenea: Dactylogyridae) parasitizing serrasalmid fish (Characiformes) from Brazil, including molecular data. Acta Parasitol.

[B032] Morey GAM, Aliano AMB, Grandez FAG (2019). New species of Dactylogyridae Bychowsky, 1933 infecting the gills of *Myloplus schomburgkii* (Jardine) and *Colossoma macropomum* (Cuvier) in the Peruvian Amazon. Syst Parasitol.

[B033] Oliveira MSB, Tavares-Dias M (2016). Communities of parasite metazoans in *Piaractus brachypomus* (Pisces, Serrasalmidae) in the lower Amazon River (Brazil). Rev Bras Parasitol Vet.

[B034] Pamplona-Basilio M, Kohn A, Feitosa VA (2001). New hosts records and description of the egg of *Anacanthorus penilabiatus* (Monogenea, Dactylogyridae). Mem Inst Oswaldo Cruz.

[B035] Pompeu PS, Godinho HP, Godinho HP, Godinho AL (2003). Águas, peixes e pescadores do São Francisco das Minas Gerais..

[B036] Ribeiro FM, Freitas PVDX, Santos EO, Sousa RM, Carvalho TA, Almeida EM (2016). Alimentação e nutrição de Pirapitinga (*Piaractus brachypomums*) e Tambaqui (*Colossoma macropomum*): revisão. Pubvet.

[B037] Santos EF, Tavares-Dias M, Pinheiro DA, Neves LR, Marinho RGB, Dias MKR (2013). Fauna parasitária de tambaqui *Colossoma macropomum* (Characidae) cultivado em tanque-rede no estado do Amapá, Amazônia oriental. Acta Amaz.

[B038] Santos JF, Muriel-Cunha V, Domingues MV (2019). New species of *Anacanthorus* (Dactylogyridae: Anacanthorinae) from the gills of *Hoplerythrinus unitaeniatus* and *Erythrinus erythrinus* (Characiformes: Erythrinidae) of the coastal drainage in the Eastern Amazon, Brazil. Zootaxa.

[B039] Silva MT, Cavalcante PHO, Santos CP (2022). Monogeneans of *Colossoma macropomum* (Cuvier, 1818) (Characiformes: Serrasalmidae) farmed in the state of Acre, Amazon (Brazil). Rev Bras Parasitol Vet.

[B040] Soberon L, Mathews P, Malherios A (2014). Hematological parameters of *Colossoma macropomum* naturally parasitized by *Anacanthorus spathulatus* (Monogenea: Dactylogiridae) in fish farm in the Peruvian Amazon. Int Aquat Res.

[B041] Van Every LR, Kritsky DC (1992). Neotropical Monogenoidea. 18. *Anacanthorus* Mizelle and Price, 1965 (Dactylogyridae, Anacanthorinae) of piranha (Characoidea, Serrasalmidae) from the central Amazon, their phylogeny, and aspects of host-parasite coevolution. J Helminthol Soc Wash.

